# Effects of race and ethnicity on hematopoietic stem cell transplant outcomes in acute myeloid leukemia: a systematic review and meta-analysis

**DOI:** 10.3389/fonc.2025.1703050

**Published:** 2025-12-11

**Authors:** Ana Melo, Shakeel Ahmed, Masuma Anzuman, Siaana Allana, Michelle Kilcoyne, Vutha Nhim, Osvaldo Padilla, Alok K. Dwivedi, Anna M. Eiring

**Affiliations:** 1 Department of Pathology, Texas Tech University Health Sciences Center El Paso, El Paso, TX, United States; 2 Department of Pathology and Genomic Medicine, Houston Methodist Hospital, Houston, TX, United States; 3 Division of Biostatistics and Epidemiology, Department of Molecular and Translational Medicine, Texas Tech University Health Sciences Center El Paso, El Paso, TX, United States; 4 Department of Biological Sciences, The University of Texas at El Paso, El Paso, TX, United States; 5 Paul L. Foster School of Medicine, Texas Tech University Health Sciences Center El Paso, El Paso, TX, United States; 6 Department of Pathology & Immunology, Baylor College of Medicine, Houston, TX, United States; 7 University of Arkansas for Medical Sciences, Washington Regional Medical Center, Fayetteville, AR, United States; 8 Department of Biomedical Informatics, Biostatistics and Medical Epidemiology, University of Missouri School of Medicine, Columbia, MO, United States

**Keywords:** acute myeloid leukemia (AML), hematopoietic stem cell transplantation (HSCT), overall survival (OS), relapse rates (RR), race/ethnicity

## Abstract

**Introduction:**

Despite the advancements in medical facilities and treatment, acute myeloid leukemia (AML) remains a significant concern. Hematopoietic stem cell transplantation (HSCT) is the preferred treatment option for AML. However, racial and ethnic disparities have a prominent impact on HSCT outcomes due to variability in treatment availability, transplant referral, donor scarcity, socioeconomic status, and other factors.

**Methods:**

In this systematic review and meta-analysis, we evaluated transplantation rates, relapse rates, and survival outcomes across racial and ethnic groups. A comprehensive search was conducted using PubMed, Embase, and Cochrane Library to screen relevant studies. Study quality was assessed using the MINORS scale and NIH tool, followed by an assessment of publication bias using the funnel plot and Egger’s test. A random-effects model was employed to evaluate the transplantation rate, relapse rate, and overall survival (OS). Cochran’s Q test and I2 statistic were utilized to assess the heterogeneity.

**Results:**

A total of 781 articles were screened, and following several stages of screening according to inclusion criteria, seven full-text articles comprising nine datasets were included in the final analysis. The pooled results for transplantation and OS were not statistically significant. However, the pooled results for relapse outcome were statistically significant for both Blacks vs. Whites (risk ratio [RR] = 1.17; 95% CI: 1.04-1.32; p=0.008; I_2_=0.0%) and Hispanics vs. Blacks (risk ratio [RR] = 0.77; 95% CI: 0.61-0.97; p=0.027; I_2_==37.3%), favoring Whites and Hispanics, respectively.

**Discussion:**

Minimizing disparities in the social determinants of health across racial and ethnic groups, along with providing equity in treatment access, are needed to improve outcomes.

**Systematic Review Registration:**

https://www.crd.york.ac.uk/PROSPERO/view/CRD420250644767, identifier CRD420250644767.

## 1 Introduction

Hematological malignancies are a diverse group of cancers originating in hematopoietic tissues, resulting from abnormal hematopoiesis. Malignancies originating in the lymphatic system are referred to as lymphomas, those originating from bone marrow plasma cells are known as plasma cell neoplasms, and those affecting both blood and bone marrow are known as leukemias (1, 2). Acute myeloid leukemia (AML) is a malignancy of blood and bone marrow (leukemia) that originates from progenitor cells or myeloid hematopoietic stem cells, leading to the excessive production of abnormal or immature white blood cells (WBCs) called blasts (3, 4). In adults, AML is the most prevalent form of leukemia, making up approximately 80% of cases. The median age of diagnosis for this condition is 67–68 years in the United States. Health outcomes for AML vary by age group, with younger patients having a 5-year overall survival (OS) rate of 30%-50%. However, OS rates drop to just 10% for elderly patients >65 years of age (5, 6). The European Leukemia Net (ELN) classified this highly heterogeneous disease in 2022 into favorable, intermediate, and high-risk groups based on mutations (7). Researchers are working to uncover the etiology of disease progression and drug resistance in AML, in order to develop effective treatment and management strategies (8, 9). Many treatment options are now available for AML, including cytotoxic therapies (e.g., Fludarabine, Clofarabine, Mitoxantrone, Etoposide, etc.), targeted therapies (e.g., Venetoclax, Enasidenib, Midostaurin, Gilteritinib, etc.), and immunotherapies (e.g., Magrolimab, donor lymphocyte infusion, CAR-T therapy, etc.) (10). The Food and Drug Administration (FDA) approved several targeted therapeutic agents, including Venetoclax, Midostaurin, Enasidenib, Gilteritinib, etc., to treat AML (11, 12). However, despite recent advances in AML prognosis and treatment, a majority of AML patients ultimately succumb to their disease.

Hematopoietic stem cell transplantation (HSCT), also known as bone marrow transplantation, is considered the most effective treatment for hematological disorders. Diseased and abnormal bone marrow cells are first eliminated through radiation and chemotherapy, and then donor bone marrow is transplanted to replace the damaged malignant cells (13, 14). HSCT can be categorized as follows: syngeneic, where the donor is an identical twin (15); autologous, where the patient’s healthy bone marrow cells are collected, purified, and reinfused into the patient (16); and allogeneic, where the donor is either a family member or an unrelated individual with HLA matching, or, in the worst case scenario, an HLA-mismatched donor (17). Syngeneic transplantation shows no risk of graft failure or graft-versus-host disease (GVHD) (15), but donor availability remains a challenge. In contrast, allogeneic transplantation carries a high risk of GVHD (18), while autologous transplantation can induce abnormal cells, leading to relapse after transplant (19). Umbilical cord blood transplantation (UCBT) is considered a form of allogeneic HSCT (allo-HSCT). The stem cells present in umbilical cord blood (UCB) are relatively immature with less plasticity and great potential for off-the-shelf use in emergencies. Transplantation of UCB exhibits a lower incidence of chronic GVHD and poses no harm to the mother and fetus. Furthermore, UCB offers flexibility in HLA matching and results in improved survival and relapse outcomes compared with other forms of allo-HSCT (20).

Despite being a complex and expensive treatment, HSCT, particularly allo-HSCT, has emerged as the preferred treatment option and a beacon of hope for patients with all types of hematological malignancies. However, it is often associated with anxiety, depression, reduced quality of life, post-traumatic stress disorder (PTSD), and other treatment-related side effects (21). Furthermore, significant disparities are evident in patient referral, transplant hospitalization, donor search and availability, supportive care, and follow-up, which ultimately results in poor treatment outcomes across ethnic and racial groups throughout the United States (22, 23). In a recent study, Fei-Zhang and colleagues demonstrated how the social determinants of health (SDoH), such as socioeconomic status, housing, household infrastructure, transportation, language barriers, race, and ethnicity negatively impact the treatment availability and survival of patients with various hematological malignancies including AML in the United States (24). Most of these disparities are prominent among Black people and Hispanics, involving disparities in referral to HSCT, utilization, survival, and relapse rates of patients (25). However, different scenarios for treatment availability and transplant outcomes are evident among non-Hispanic White (NHW) and Asian individuals (26).

We hypothesized that race and ethnicity are associated with differences in HSCT outcomes among patients with AML. To test this hypothesis, we conducted a systematic review and meta-analysis to evaluate the transplantation rates, relapse rates, and overall survival (OS) of AML patients from different racial and ethnic groups, including Hispanics, Black people, and Asians, compared with non-Hispanic Whites following HSCT. Furthermore, direct comparisons between Hispanic and Black individuals were also conducted.

## 2 Materials and methods

### 2.1 Defining the research question

The research question for this systematic review and meta-analysis was formulated using the standardized POSE framework (27). It includes: Population, (P) = AML following HSCT; Outcome, (O) = Transplantation rate, relapse rate, and OS; Study Design, (S) = Cohort or intervention follow-up studies; and Exposure, (E) = Hispanic, Black, and Asian patients vs. non-Hispanic White patients; Hispanic vs. Black patients. In addition, the included original studies must be conducted on US populations so that racial/ethnic definitions are consistent across included studies. This study was conducted following the guidance of the Cochrane Collaboration and directed by PRISMA (Preferred Reporting Items for Systematic Reviews and Meta-Analyses) (28). Additionally, this study was registered with PROSPERO (the International Prospective Register of Systematic Reviews), and its identification code is CRD420250644767 (29). Transplantation rate in this systematic review and meta-analysis was defined as the number of AML patients who underwent HSCT among the total diagnosed patients with AML during a specific period within a specific study cohort (30, 31).

### 2.2 Literature search strategy

Several keywords were carefully defined to search for and identify the literature and data of interest. Two authors (Ana Melo and Vutha Nhim) independently and systematically searched the PubMed, Cochrane Library, and Embase databases in August 2024 and again in January 2025, using the keywords as: “transplant” OR “transplantation”, “acute myeloid leukemia” OR “AML” OR “blood cancer”, and “Hispanic” OR “race” (14). Furthermore, the cohort enrollment periods among the included studies ranged from 1989 to 2017 and the details of cohort enrollment times are presented in **Table 1**.

**Table 1:** Summary of the study populations.

Study	Data source	Ethnicity	Population	Population size	Population characteristics	Time of enrollment
Gramatges et al., 2017 (43)	Texas Children’s Cancer Center (TCCC)	Hispanic, and non-Hispanic White (NHW)	Children in the age group of 0 to 18 years	129 patients (72 Hispanics and 57 NHWs)	Diagnosed with *de novo* AML	1^st^ January 1998 to 31^st^ December 2015
Aplenc et al. (CCG 2891), 2006 (44)	Children’s Oncology Group	Black, White, Hispanic, and Asian	Children to 21 years old adults	791 patients (114 Hispanics, 558 White people, 94 Black people, and 25 Asians)	Diagnosed with AML	1989 to 1995
Aplenc et al. (CCG 2961), 2006 (44)	Children’s Oncology Group	Black, White, Hispanic, and Asian	Children to 21 years old adults	850 patients (157 Hispanics, 583 White people, 84 Black people, and 26 Asians)	Diagnosed with AML	1996 to 2002
Blue et al., 2023 (46)	Center for International Blood and Marrow Transplant Research (CIBMTR)	non-Hispanic White, non-Hispanic Black, Hispanic, and Asian	Individuals over 18 years of age	5,473 patients (516 Hispanics, 4,385 NHWs, 338 Black people, and 234 Asians)	Diagnosed with acute myeloid leukemia (AML), acute lymphoblastic leukemia (ALL), chronic myeloid leukemia (CML), and myelodysplastic syndromes (MDS)	2007 to 2017
Baker et al., 2005 (42)	Center for International Blood and Marrow Transplant Research (CIBMTR)	Black, White, Asian, and Hispanic	Patients of all ages	3,028 patients (237 Hispanics, 2,418 White people, 251 Black people, and 122 Asians)	Diagnosed with AML, CML, or ALL	1990 to 2000
Ballen et al., 2012 (47)	Center for International Blood and Marrow Transplant Research (CIBMTR)	Black, White, and Hispanic	Children and adults	885 patients (128 Hispanics, 612 White people, and 145 Black people)	Diagnosed with AML, CML, ALL, and MDS	1995 to 2006
Patel et al., 2015 (26)	California Cancer Registry (CCR)	White, Black, Hispanic, Asian/Pacific Islanders (API)	Patients of all ages	11,084 patients (1,936 Hispanics, 7,381 White people, 603 Black people, and 1,164 API)	Diagnosed with AML in California	1998 to 2008
Ballen et al. (Adults), 2024 (45)	Center for International Blood and Marrow Transplant Research (CIBMTR)	Black, White, Asian, and Latinx (Hispanic)	US patients, age ≥18 years	1,705 patients (235 Hispanics, 1,132 White people, 199 Black people, and 139 Asians)	Diagnosed with acute myeloid leukemia, acute myelodysplasia, or acute lymphoid leukemia.	2007 to 2017
Ballen et al. (Pediatrics), 2024 (45)	Center for International Blood and Marrow Transplant Research (CIBMTR)	Black, White, Asian, and Latinx (Hispanic)	US patients, aged between 1 to <18 years	807 patients (261 Hispanics, 385 White people, 123 Black people, and 38 Asians)	Diagnosed with acute myeloid leukemia, acute myelodysplasia, or acute lymphoid leukemia.	2007 to 2017

### 2.3 Eligibility criteria and data extraction

All duplicate records or articles were identified and eliminated from the list electronically. Two authors (Ana Melo and Vutha Nhim) independently screened all the records according to title and abstract, excluded the irrelevant records, and extracted some full-text articles. The authors then reviewed the full-text articles, selected those most relevant to our study, and eliminated the remaining ones. For the final screening, predefined exclusion criteria were applied, including the exclusion of records involving populations outside the target group, articles lacking control groups, articles with insignificant outcomes, and articles written in languages other than English, Spanish, or Portuguese. The authors regularly shared updates on their work to maintain consistency throughout the research and avoid disagreements (32). There were no discrepancies between the two reviewers; therefore, Cohen’s kappa statistic (κ) for agreement between authors was not calculated. We extracted data on study authors, year of publication, study sample criteria, study design, racial and ethnic comparisons, follow-up duration, sample size for each racial or ethnic group, average age, gender distribution, comorbidities, number of transplants, relapse rate, and survival status post-transplantation. All extracted data were summarized using Microsoft Excel 2021.

### 2.4 Quality assessment and publication bias

Two authors independently assessed the quality of non-randomized observational studies using the Methodological Index for Non-Randomized Studies (MINORS) tool. MINORS is a rigorous and validated quality assessment tool comprising 12 items for the assessment of methodological standards in non-randomized observational studies. Of these, the first eight items are applicable to both comparative and non-comparative studies. However, the last four items are appropriate only for studies comprising two or more groups. Additionally, each of these items was graded from 0 to 2, indicating not reported (0), reported but not adequate (1), and reported adequately (2) (33–35). For assessing the quality of randomized clinical trials, we used the National Institutes of Health (NIH) quality assessment tool. A total of fourteen criteria were assessed for each study, and the overall quality was rated as good, fair, or poor (36, 37).

Publication bias is a major concern in systematic reviews and meta-analyses within medical research. To evaluate the publication bias of statistically significant studies, a funnel plot and Egger’s test were conducted (38, 39).

### 2.5 Statistical analysis

For each study, we extracted the frequency and risk or odds or hazard ratio for transplantation, post-transplant survival, and relapse rate, along with the corresponding 95% confidence intervals (CI) or standard error. In this study, p-values less than 0.05 (p<0.05) were considered statistically significant. Cochran’s Q-test and I^2^ statistic were used to assess heterogeneity, with a p-value of 0.1 in the Q-test indicating a statistically significant heterogeneity (32, 40). *A priori*, the DerSimonian and Laird (D-L) random-effects model was used to obtain the pooled effect size for all outcomes (28). Racial and ethnic differences in transplantation rate were summarized using a pooled odds ratio (OR), while survival status and relapse rate were summarized using a pooled risk ratio (RR), each with a 95% confidence interval (CI). Sensitivity analyses were conducted in case of significant heterogeneity according to pediatric and adult studies. Forest plots were constructed to visually assess statistical differences in transplantation rate, relapse rate, and OS outcomes among AML patients of different races and ethnicities (i.e., Hispanics, Asians, Black people, and non-Hispanic Whites) following HSCT. All statistical analyses were carried out using Stata 17 (41).

## 3 Results

### 3.1 Search results and study selection

**Figure 1:**
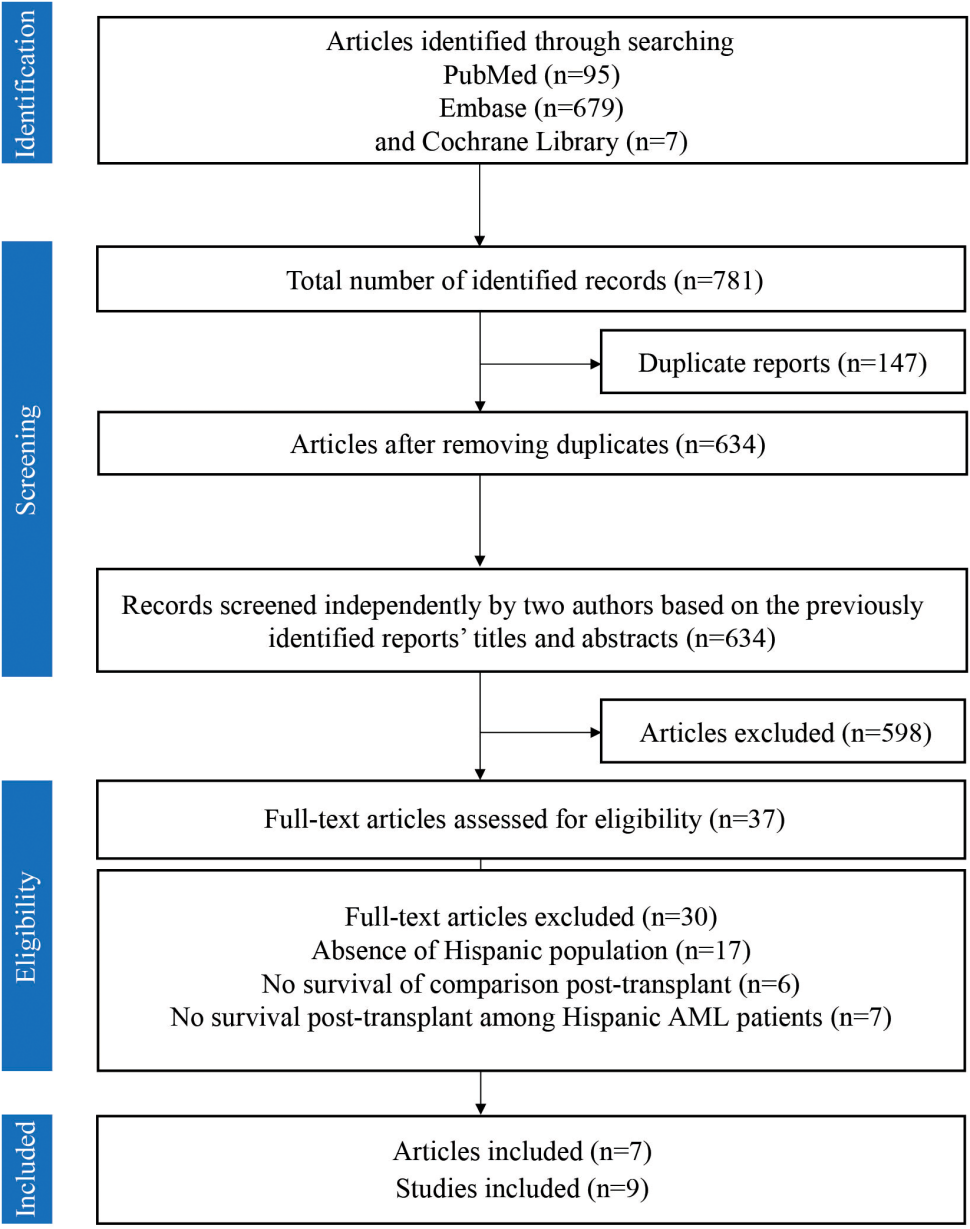
PRISMA flow diagram illustrating the screening and selection process of studies. The flow diagram illustrates the screening and selection process utilized in the present study.

A total of 781 articles were initially identified for this systematic review and meta-analysis, including 95 articles from PubMed, 679 from Embase, and 7 from the Cochrane Library. After removing 147 duplicate articles, two authors screened the remaining 634 articles. Excluding 597 irrelevant articles, 37 full-text articles were subsequently extracted and screened for eligibility. Among these, 30 articles were excluded based on the predefined exclusion and inclusion criteria. Finally, 7 articles comprising 9 studies were included in the meta-analysis. **Figure 1** illustrates the study selection flow diagram, following the PRISMA framework.

### 3.2 Characteristics of eligible studies and study populations

Seven retrospective and two prospective datasets were selected and included in this meta-analysis (**Supplementary Table S1**). The data from these studies were limited to the United States and published between 2005 and 2024. Among the nine studies, one recorded data for 3,028 patients diagnosed with AML, ALL, or CML from various ethnic groups, including Hispanics, Black people, Asians, and non-Hispanic Whites across all age groups. These patients received HLA-identical sibling HSCT (allo-HSCT) (42). The study conducted by Gramatges and colleagues reviewed data from 129 *de novo* AML patients (ages ranging from 0 to 18 years) who were either Hispanic or non-Hispanic White (43). Another study included patients of Hispanic, non-Hispanic White, Black, and Asian into two groups, CCG 2891 with 791 *de novo* AML patients and CCG 2961 with 850 *de novo* AML patients (44). Patel et al., collected data from 11,084 patients, comprising Black people, White people, Hispanics, and Asian/Pacific Islanders, who were diagnosed with AML in California. These data were collected from the California Cancer Registry (CCR) (26). Two of the most recent studies conducted by Ballen and colleagues collected data from 1,705 adult patients and 807 pediatric patients across all ethnicities, who underwent UCB transplantation for AML, MDS, or ALL and were registered in the Center for International Blood and Marrow Transplant Research (CIBMTR) (45). The remaining two studies also used the CIBMTR database to identify and collect data on patients diagnosed with AML, CML, ALL, or MDS (46, 47). Among these, one study included 5,473 patients aged over 18 years from all ethnicities who survived at least one year after transplantation (46). Another study included 885 adults and children from Black, White, and Hispanic patients receiving UCB transplantation as an alternative to HSCT (47). A summary of the study populations is presented in **Table 1** and a summary of study characteristics including outcomes of transplantation is presented as **Supplementary Table S1**.

### 3.3 Quality (risk of bias) assessment and publication bias

**Figure 2:**
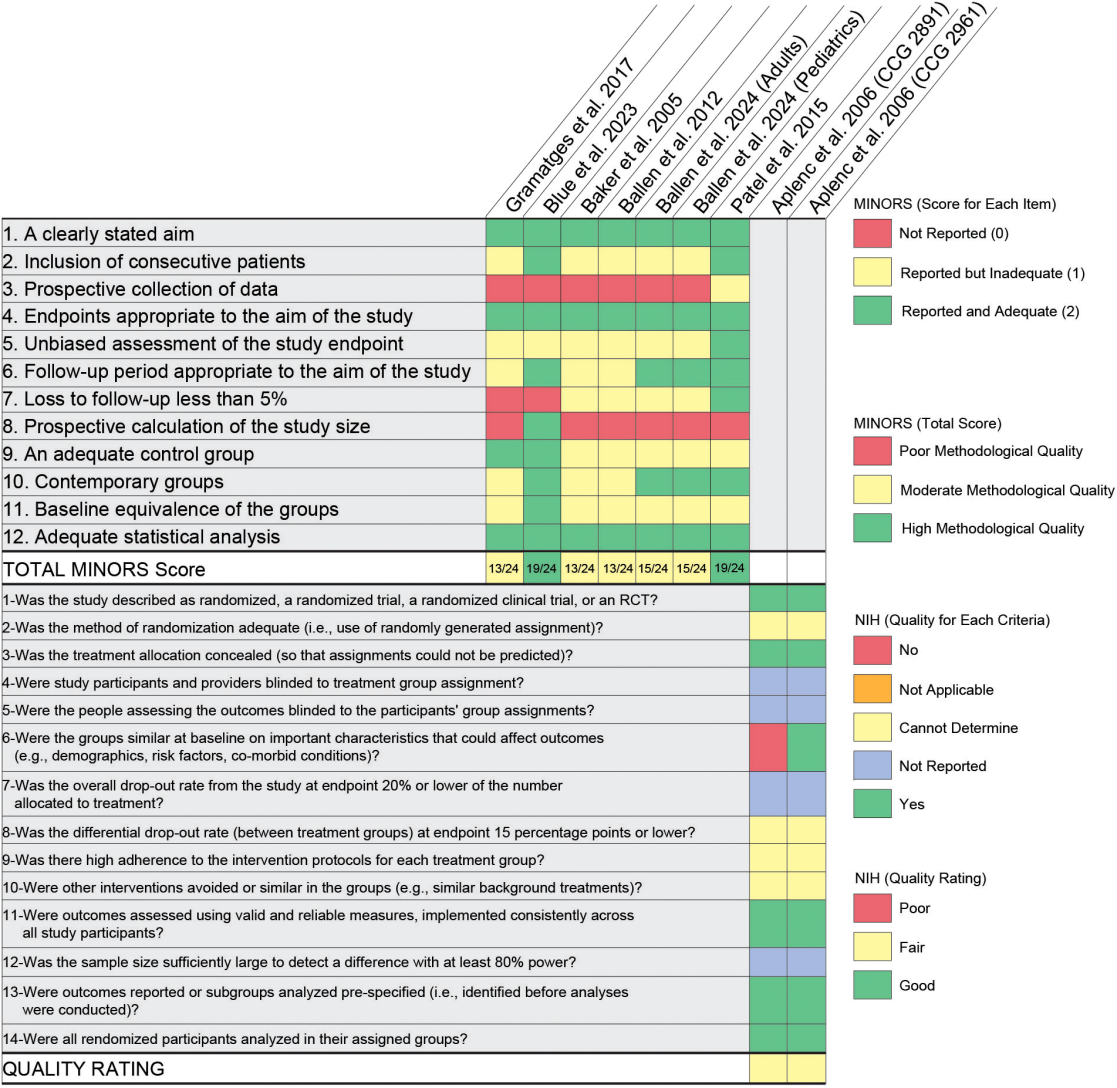
Heatmap illustrating the quality assessment of the included studies. The heatmap shows the quality assessment of the studies using both the MINORS and the NIH quality assessment tools. MINORS: ‘not reported (0)’ is indicated in red, ‘reported but inadequate or partially reported (1)’ in yellow, and ‘reported and adequate (2)’ in green color. NIH: ‘yes’ is indicated by green, ‘no’ by red, ‘not applicable (NA)’ by orange, ‘cannot determine’ by yellow, and ‘not reported’ by blue color.

**Figure 2** illustrates the quality assessment of the included studies using both the MINORS and the NIH quality assessment tools. For the studies assessed with the MINORS tool, ‘not reported (0)’ is indicated in red, ‘reported but inadequate or partially reported (1)’ in yellow, and ‘reported and adequate (2)’ in green color. All the non-randomized observational studies exhibited moderate methodological quality, except for the study conducted by Blue et al. (46), and Patel et al. (26), which demonstrated high methodological quality (total score: 19/24).

In contrast, the answer to the questions asked in the NIH tool for assessing the quality of randomized control trials, ‘yes’ is indicated by green, ‘no’ by red, ‘not applicable (NA)’ by orange, ‘cannot determine’ by yellow, and ‘not reported’ by blue color. The final quality rating for both studies was rated as ‘fair’ (44).

Additionally, studies comparing relapse outcomes in Black people vs. White people and Hispanics vs. Black people exhibited no evidence of publication bias. However, publication bias was present in the study comparing transplantation between Hispanics and Black people (**Supplementary Table S2**; **Supplementary Figure S1**).

### 3.4 Transplantation rate

**Figure 3:**
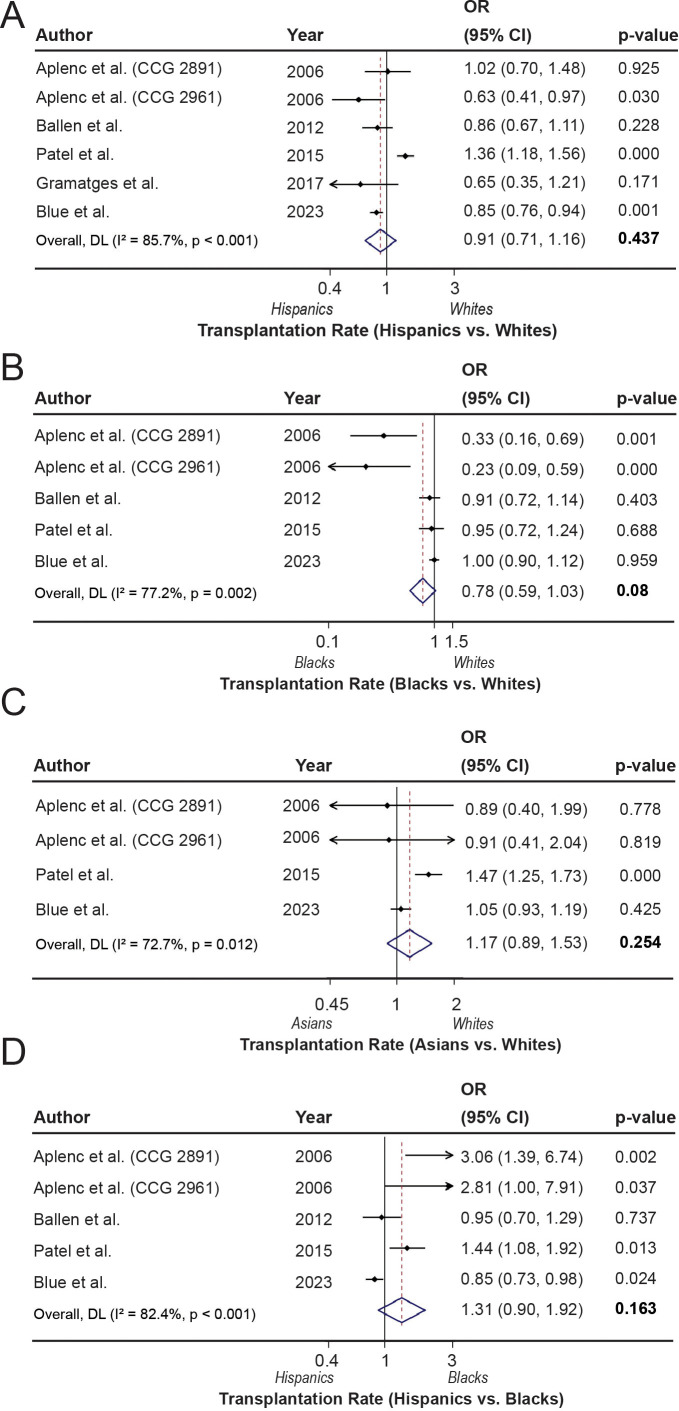
Hematopoietic stem cell transplantation rates across racial and ethnic groups. **(A–D).** The forest plots show the transplantation rates comparing Hispanic with White (reference) patients **(A)**, Black with White (reference) patients **(B)**, Asian with White (reference) patients **(C)**, and Hispanic with Black (reference) patients **(D)**.

P values for the individual studies are presented in **Supplementary Table S3**. The rate of HSCT among AML patients across racial and ethnic groups was assessed in this study and illustrated in **Figure 3**. The overall effect size for the transplantation rate was not found to be different between Hispanic and White patients (OR = 0.91; 95% CI, 0.71-1.16; p=0.437, I^2^ = 85.7%) as well as between Asian and White AML patients (OR = 1.17; 95% CI, 0.89-1.53; p= 0.254, I^2^ = 72.7%) (**Figures 3A, C**). These differences remained insignificant even with stratified analysis by study population, with high heterogeneity (**Supplementary Tables S5, S6**). However, Black patients tended to have a lower rate of transplantation compared with White patients (OR = 0.78; 95% CI, 0.59-1.03; p= 0.08, I^2^ = 77.2%) (**Figure 3B**) with a marked effect size in pediatric patients (OR = 0.45; 95%CI: 0.17-1.16, p=0.098, I^2^ = 85.1%) (**Supplementary Table S5**). These non-significant differences may be potentially due to the low number of studies with large heterogeneity. Similarly, a 31% higher transplantation rate was associated with Hispanics compared to Black people (OR = 1.31; 95% CI, 0.90-1.92; p=0.163, I^2^ = 82.4%), although the association was not statistically significant (**Figure 3D**). However, after excluding older AML patients, the transplantation rate was found to be significantly higher in Hispanics (OR = 2.02; 95% CI: 1.15-3.54; p=0.014) compared with Black people, with a considerably lower and more acceptable heterogeneity (I^2^ = 53.1%), while these differences were insignificant when excluding studies with pediatric patients (**Supplementary Tables S5, S6**).

### 3.5 Relapse rates

**Figure 4:**
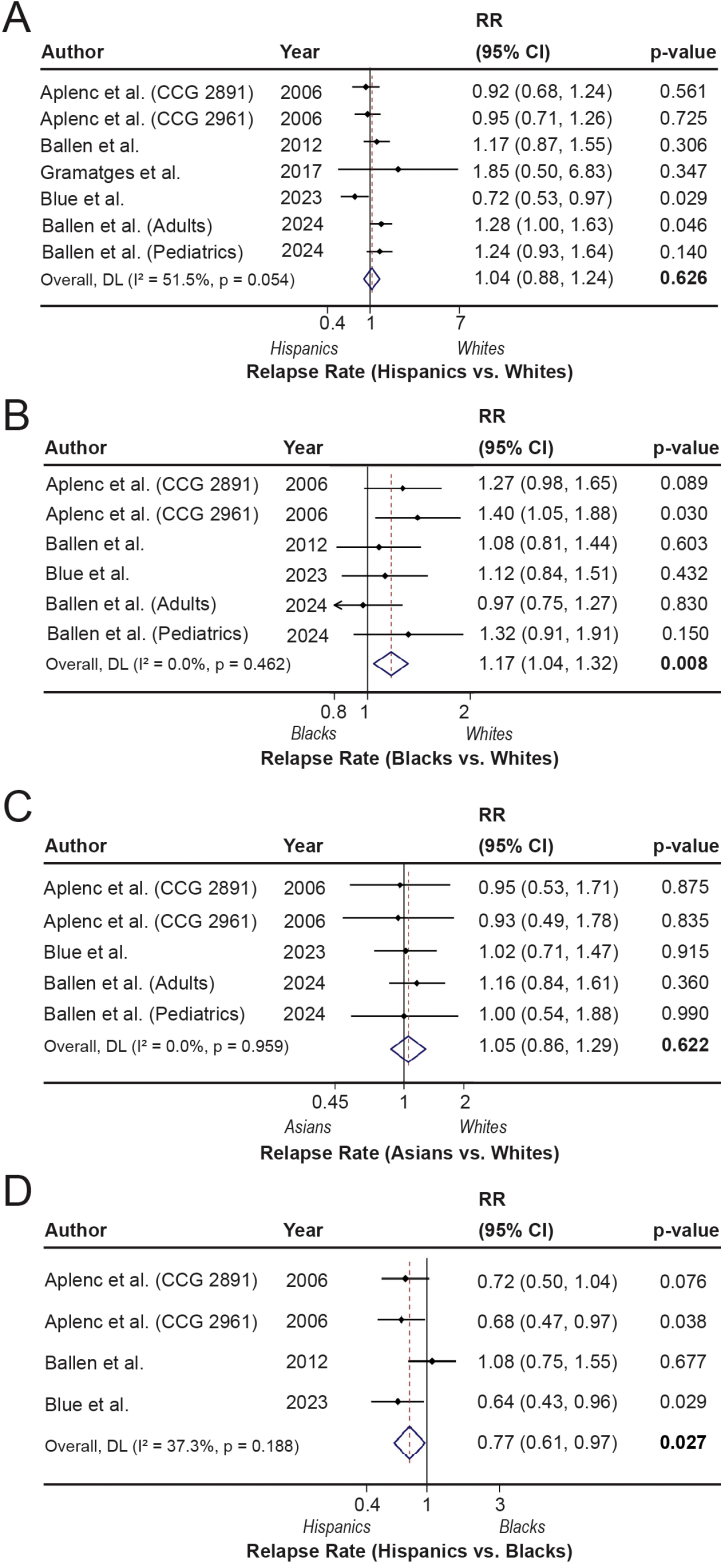
Relapse rates (RR) across racial and ethnic groups following hematopoietic stem cell transplantation (HSCT). **(A-D).** The forest plots show the relapse rates comparing Hispanic with White (reference) patients **(A)**, Black with White (reference) patients **(B)**, Asian with White (reference) patients **(C)**, and Hispanic with Black (reference) patients **(D)**.

We also compared relapse rates, defined as disease recurrence and progression, across different racial and ethnic groups, including Asians, Black people, and Hispanics, relative to non-Hispanic Whites, as well as between Hispanics and Black people. The results of these comparisons are illustrated in **Figure 4**. The relapse rate was not significantly associated with Hispanics (RR = 1.04; 95% CI, 0.88-1.24; p=0.626, I^2^ = 51.5%) or Asians (RR = 1.05; 95% CI, 0.86-1.29; p=0.622, I^2^ = 0.0%) compared with White people (**Figures 4A, C**). These differences were unchanged in heterogeneity evaluation analyses (**Supplementary Tables S5, S6**). However, a significantly higher relapse rate was found in Black people (RR = 1.17; 95% CI, 1.04-1.32; p=0.008) compared with White people, without any heterogeneity (I^2^ = 0.0%) (**Figure 4B**). The overall pooled results remained statistically significant, with no heterogeneity after excluding older patients (RR = 1.33; 95% CI: 1.12-1.58; p=0.001) (**Supplementary Table S5**). Moreover, a favorable relapse outcome was associated with Hispanics compared to Black people (RR = 0.77; 95% CI, 0.61-0.97; p=0.027) without significant presence of heterogeneity (I^2^ = 37.7%) (**Figure 4D**). These associations were more pronounced among pediatric patients with no heterogeneity (RR = 0.70; 95% CI: 0.54-0.90; p=0.006) (**Supplementary Table S5**).

### 3.6 Overall survival

**Figure 5:**
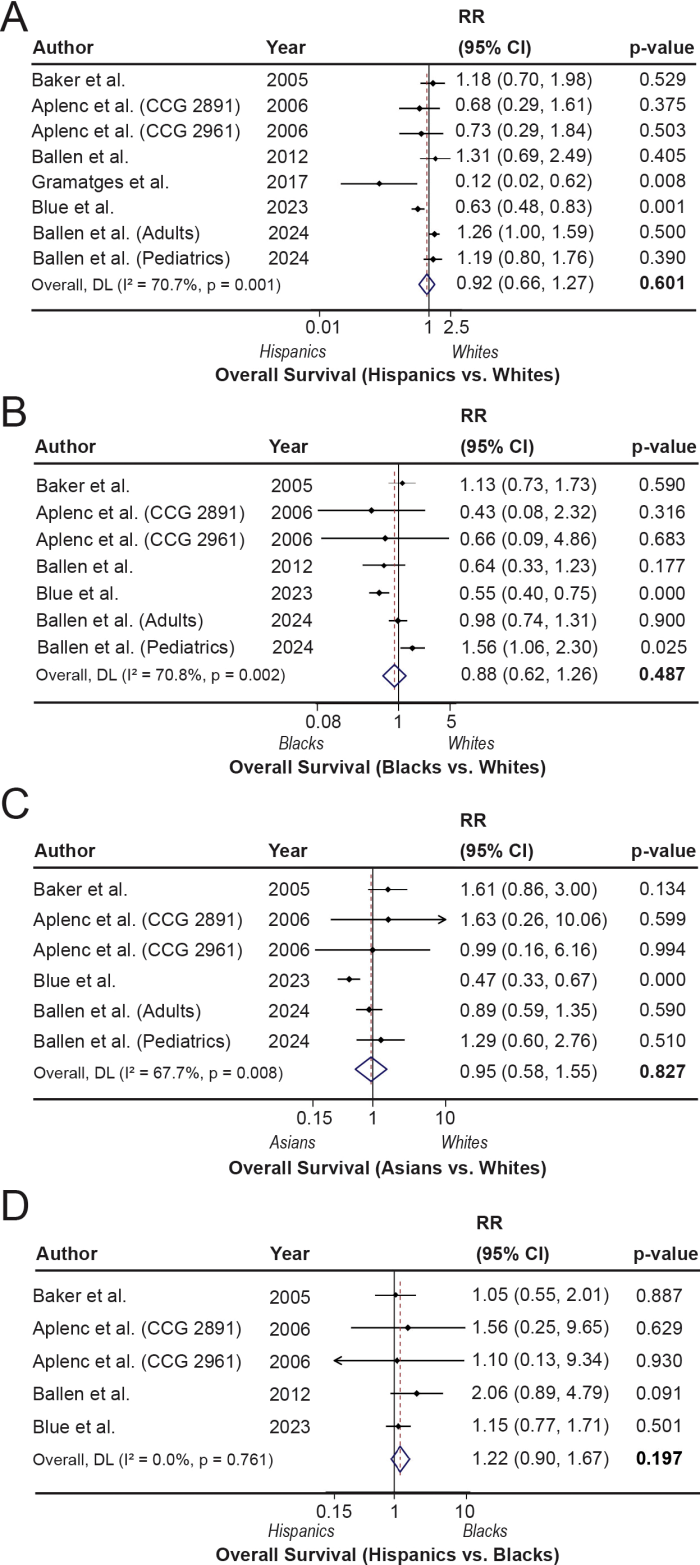
Overall survival (OS) across racial and ethnic groups following hematopoietic stem cell transplantation (HSCT). **(A–D)**. The forest plots show the overall survival (OS) rates comparing Hispanic with White (reference) patients **(A)**, Black with White (reference) patients **(B)**, Asian with White (reference) patients **(C)**, and Hispanic with Black (reference) patients **(D)**.

**Figure 5** illustrates the survival outcomes across different racial groups relative to White people. Compared with White people, Hispanics (RR = 0.92; 95% CI, 0.66-1.27; p=0.601, I^2^ = 70.7%), Black people (RR = 0.88; 95% CI, 0.62-1.26; p=0.487, I^2^ = 70.8%), or Asians (RR = 0.95; 95% CI, 0.58-1.55; p=0.827, I^2^ = 67.7%) did not show any difference in OS outcome in all studies (**Figures 5A–C**). No differences in survival outcomes were noticed even after exploring heterogeneity owing to mixed study populations (**Supplementary Tables S5, S6**). Although not statistically significant, Black patients had poor OS compared with Hispanics (RR = 1.22; 95% CI, 0.90-1.67; p=0.197), with no heterogeneity (**Figure 5D**), particularly in studies in predominantly older patients (RR = 1.22; 95% CI: 0.89-1.67; p=0.22) without any heterogeneity (**Supplementary Table S6**). In addition, Asian AML patients also demonstrated a higher risk of mortality compared to White patients without any heterogeneity after excluding studies with older patients (RR = 1.45; 95% CI: 0.92-2.27; p=0.111), yet the difference was not statistically significant (**Supplementary Table S5**).

## 4 Discussion

AML is a type of hematological disorder, and the malignant growth of progenitor or hematopoietic stem cells is the hallmark of this malignancy (48). We aimed to assess the impact of race and ethnicity on transplantation rates and outcomes, particularly overall survival and relapse rates, following hematopoietic stem cell transplantation. Although our study did not find any statistically significant differences in transplantation rates and overall survival among different racial and ethnic groups, a better relapse outcome with statistical significance was noticed among non-Hispanic White individuals compared to their Black counterparts without any heterogeneity. Hispanics also exhibited a lower relapse rate when compared to Black people. In stratified analysis by study population, a lower transplantation rate was associated with Black people compared to Hispanics. Moreover, a trend association towards poor OS in Black people and Asians compared to White people was noticed in our meta-analysis.

To assess the robustness of our findings, sensitivity analyses were conducted by excluding studies with only older AML patients and those restricted to pediatric AML patients. Substantial heterogeneity (I^2^>50%) was observed for transplantation and OS outcomes (except for OS in Hispanics vs. Black people, I^2^ = 0.0%). In contrast, relapse outcomes demonstrated acceptable heterogeneity (I^2^<50%) among the studies. The exclusion of studies involving older patients for transplantation resulted in considerably acceptable heterogeneity for Asians vs. White people (I^2^ = 24.1%) and Hispanics vs. Black people (I^2^ = 53.1%). Similarly, acceptable heterogeneity was obtained for OS (I^2^ ≤ 54%) and relapse (I^2^<9%) following the exclusion of studies involving older AML patients. However, the exclusion of studies involving pediatric AML patients resulted in higher heterogeneity in most of the analyses (I^2^>50%). These findings demonstrate that pediatric AML patients are more homogeneous and thus contribute to consistent results. Additionally, age is not the only factor contributing to heterogeneity. The number of included studies, study design, and methodological quality also play significant roles. After excluding key heterogeneity sources such as older and pediatric patients, most of the overall pooled effect remained consistent. However, the transplantation rate in Hispanics vs. Black people became statistically significant after excluding older patients, while relapse in Black people vs. White people and Hispanics vs. Black people became statistically nonsignificant after excluding pediatric patients.

Theresa Hahn and colleagues conducted a cohort study and found that allogeneic transplantation rates for acute myeloid leukemia and myelodysplastic syndrome were the same among White people, Hispanics, and other races. However, Hispanics received more transplants than Black people, which aligns with our study finding (25). B. K. Hamilton et al. (49), and J. Bierenbaum et al. (50), observed no statistically significant difference in OS between White people and Black people following HSCT after adjusting for some factors, which supports our study findings. Black people, particularly adolescents and young adults often experience delays in disease diagnosis and healthcare access, contributing to higher rates of early deaths. Furthermore, higher rates of mutations in *ASXL1* and *BCOR* genes, as well as lower frequency of mutations in *NPM1* and biallelic *CEBPA* genes tend to be responsible for treatment resistance and worse relapse in Black people than White people (51). Over the years, several studies have found that “Hispanic paradox” surprisingly gives health benefits and better outcomes to U.S. Hispanic populations (52). A study conducted by Ashley and colleagues reported improved post-liver transplantation outcomes in Hispanics compared to Black people (53). Recently, two studies conducted in Canada highlighted racial disparities in HSCT outcomes. A study conducted by Mohapatra et al., reported poor overall survival and higher non-relapse mortality among South Asian patients compared with White people and East Asians (54). In contrast, Herrity et al., observed no statistically significant difference in transplant outcomes among patients who underwent allogenic HSCT (55). These findings support our major observations regarding relapse outcomes, where Black people experienced poor relapse rates compared to White people and Hispanics.

Many studies have attempted to uncover the non-biological factors contributing to these disparities; however, the causes remain elusive to this day (23, 56, 57). Cultural differences among ethnic groups, lack of healthcare facilities, delayed diagnosis, and insurance-related issues have been identified as prominent causes in several studies. Furthermore, factors such as lower socioeconomic conditions, neighborhood poverty, donor scarcity, biological and genetic factors, insurance status, age, sex, and others are likely to influence disease diagnosis, progression, treatment, availability of HSCT, response, and ultimately, survival and relapse outcomes (42, 46). Additionally, the overrepresentation of White individuals and the underrepresentation of ethnic minorities in HSCT referrals, along with geographic barriers and the limited availability of HLA-matched donors, create significant challenges for minorities in accessing this treatment (22). Language can also be a barrier to access, utilization, and outcomes of HSCT. In the United States, patients speaking non-English languages are less likely to be screened for hematological malignancies, resulting in delayed diagnosis. On top of that, their limited understanding of disease severity and other factors due to the language barrier makes them less eligible for receiving high-quality healthcare. As a result, these patients have lower rates of undergoing HSCT, a higher incidence of GVHD, and worse outcomes compared to English-speaking patients (58).

To the best of our knowledge, this is the first systematic review and meta-analysis investigating transplantation rates, overall survival, and relapse rate across United States racial and ethnic groups among patients who received HSCT. Landry previously conducted a systematic review of studies examining access to HSCT and transplant outcomes. The review identified major disparities, including donor shortage, HLA mismatch, delay in referral, and worse post-transplant outcomes in minority groups, particularly Black and Hispanic leukemia patients. However, the review didn’t specifically compare transplantation rates and transplant outcomes across racial groups (14). We included a large cohort of Hispanic, Black, White, and Asian patients diagnosed with acute myeloid leukemia and enrolled in treatment between 1989 and 2017 in the United States. Apart from evaluating the transplantation rate, survival and relapse outcomes, we assessed the sensitivity of these comparisons by conducting separate analyses excluding studies with only pediatric and older patients, which substantially reduced heterogeneity in the pooled estimates. We included both prospective and retrospective studies with clear information about follow-up records post-transplantation.

There are several limitations of the study addressing the research question. Only seven articles comprising nine datasets aligned with our research hypothesis and met the inclusion criteria. As a result, a limited amount of data was available for conducting the systematic review and meta-analysis to observe the differences in health outcomes across racial and ethnic groups. Among the included studies, seven studies were retrospective and had non-randomized collection of data, while only two prospective studies had randomized data collection. Retrospective studies may introduce potential selection and recall bias. These studies do not collect data in real-time, and patients are non-randomly assigned to the groups, which may result in reporting bias and missing data. Although the methodological quality of the two studies was high, seven studies exhibited moderate or fair methodological quality, raising questions about their quality, reliability, and validity. All the studies reported unadjusted effects and some reports both adjusted and unadjusted that may affect the overall effect size. Apart from these, the population or sample size, characteristics and age of the patients of each study were different, which might have contributed to non-significant results in the meta-analysis. Owing to the limited number of original studies meeting the eligibility criteria and the limited reporting of baseline characteristics by race/ethnic groups, we were unable to perform subgroup analyses by conditioning intensity, HLA matching, and transplant modalities. Among the included articles, four articles comprising five studies extracted data from the Center for International Blood and Marrow Transplant Research. Although these studies utilized the same data source, they were conducted by independent groups and can be considered distinct based on their sample sizes, eligibility criteria and data collection periods. However, inclusion of overlapping patients cannot be entirely ruled out, which may have introduced a potential risk of bias and influenced the overall effect sizes. As our inclusion criteria were limited to studies involving patients from the United States, none of the included studies reported data on transplantation rates and outcomes from low or middle-income countries. Furthermore, the nutritional status of patients is known to influence post-transplant outcomes. However, the studies included in this review did not report on patients’ nutritional profiles. Future research could replicate our study protocol in other settings to evaluate these parameters to enhance the global understanding of hematopoietic stem cell transplantation outcomes. Non-relapse mortality (NRM) and treatment-related mortality (TRM) reflect the differences in treatment access, complications and comorbidities. However, we were unable to analyze these parameters because the included studies did not report NRM or TRM data stratified by ethnic groups.

Our study findings once again demonstrate that racial and ethnic minorities continue to experience disparities in access to hematopoietic stem cell transplantation and in relapse rates. Necessary steps should be taken to minimize these inequities. At present, several transplant communities and national policies, such as the Affordable Care Act (ACA), are working together to improve access to transplantation for minority populations. The gap between private and public health insurance is trying to be reduced. Apart from improving health insurance coverage, the ACA aims to provide essential health benefits and support preventive care for patients, regardless of their racial and ethnic background. Transplant communities are also taking the initiative to offer better training to healthcare providers, and to educate underserved populations about the financial aspects of transplantation, the transplant referral, and selection process. Additionally, efforts are being made to increase the donor pool by recruiting living or marginal donors and exploring alternative donor sources (13, 59, 60). Furthermore, measures should be implemented to increase awareness and education among younger minority populations regarding transplantation and their accessibility.

## Data Availability

The original contributions presented in the study are included in the article/**Supplementary Material**. Further inquiries can be directed to the corresponding author.
