# Supplementary material for: Effects of race and ethnicity on hematopoietic stem cell transplant outcomes in acute myeloid leukemia: a systematic review and meta-analysis
Source: Front Oncol. 2025 Dec 11;15:1703050. doi: 10.3389/fonc.2025.1703050 (PMC12738330; doi:10.3389/fonc.2025.1703050)
Supplement: Supplementary file 1 [file DataSheet1.pdf]

SUPPLEMENTAL MATERIAL

Effects of Race and Ethnicity on Hematopoietic Stem Cell Transplant (HSCT) Outcomes in Acute Myeloid Leukemia (AML): A Systematic Review and Meta-Analysis

Ana Melo[1#], Shakeel Ahmed[2#], Masuma Anzuman[3#], Siaana Allana[4], Michelle Kilcoyne[4,5], Vutha Nhim[4,6], Osvaldo Padilla[1,4], Alok K. Dwivedi[2,4,7], Anna M. Eiring[3]*

[1]Department of Pathology, Texas Tech University Health Sciences Center El Paso, El Paso, TX, USA; [2]Division of Biostatistics and Epidemiology, Department of Molecular and Translational Medicine, Texas Tech University Health Sciences Center El Paso, El Paso, TX, USA; [3]Department of Biological Sciences, The University of Texas at El Paso, El Paso, TX, USA.[4]Paul L. Foster School of Medicine, Texas Tech University Health Sciences Center El Paso, El Paso, TX, USA; [5]Department of Pathology & Immunology, Baylor College of Medicine, Houston, TX, USA; [6]University of Arkansas for Medical Sciences, Washington Regional Medical Center, Fayetteville, AR, USA; [7]Department of Biomedical Informatics, Biostatistics and Medical Epidemiology, University of Missouri School of Medicine, Columbia, MO, USA.

*Anna M. Eiring, Ph.D.: ameiring@utep.edu

#These authors contributed equally to this work.

Running Title: HSCT Outcomes in AML

Keywords: Acute myeloid leukemia (AML), Hematopoietic stem cell transplantation (HSCT), Overall survival (OS), Relapse rates (RR), Race/ethnicity.

Supplementary Tables

Study	Follow-up	Study Design	Effect Estimates	Result/Conclusion
**Gramatges et al., 2017** (41)	Variable	Retrospective	Both adjusted and unadjusted effects	Hispanics had worse survival and relapse rates compared to non-Hispanic Whites (NHWs).
**Aplenc et al. (CCG 2891), 2006**(42)	Variable- 6 years	Prospective	Both adjusted and unadjusted effects	Hispanic and Black children exhibited worse survival and relapse outcomes. Black children experienced scarcity in donor availability, had prolonged hospital stays, and higher infection-related mortality.
**Aplenc et al. (CCG 2961), 2006**(42)	Variable-3 years	Prospective	Both adjusted and unadjusted effects	Black and Hispanic children had lower overall survival and event-free survival (EFS), along with higher infection-related mortality. Additionally, Black children experienced lower donor availability. However, remission rates were similar across the ethnic groups.
**Blue et al., 2023**(44)	1-year survival after transplantation	Retrospective	Adjusted effects	Asian and Black patients showed both lower survival and relapse rates compared to NHWs. However, Hispanics had lower survival and slightly better relapse rates.
**Baker et al., 2005**(40)	Variable	Retrospective	Both adjusted and unadjusted effects	Hispanics exhibited lower survival rates and a higher risk of treatment failure. Furthermore, no significant difference in acute or chronic graft-versus-host disease (GVHD), treatment-related mortality (TRM), or relapse was observed among patients across ethnic groups.
**Ballen et al., 2012**(45)	3 years of overall survival	Retrospective	Adjusted effects	Blacks showed lower survival rates than Whites; however, better survival was observed when higher doses of cells were used. Relapse was the leading cause of death across all ethnicities and Hispanic patients had the highest mortality (47%), compared to White (37%) and Black (38%). Additionally, 25% of Hispanics and Whites died due to infection, while 15% of Blacks died due to organ failure. Minimal GVHD risk was observed across racial and ethnic groups.
**Patel et al., 2015**(26)	5 years	Retrospective	Adjusted effects	Black patients were less likely to receive both chemotherapy and hematopoietic stem cell transplantation, while Hispanics had limited access to HSCT. In contrast, API patients did not experience any treatment disparities and demonstrated better survival than Hispanics and Blacks. However, increasing access to treatment improved survival across all racial and ethnic groups.

**Ballen et al. (Adults), 2024**(43)	Short-term (1 and 2 years) and long-term (5 years)	Retrospective	Both adjusted and unadjusted effects	Hispanic adults had worse overall survival, though survival improved over time across all groups. However, the rates of graft vs. host disease were similar across ethnic groups. High poverty levels decreased OS due to treatment-related mortality (TRM). Insurance type and education had no impact on post-transplant outcomes. Receiving umbilical cord blood units from donors of the same race or ethnicity did not improve survival.
**Ballen et al. (Pediatrics), 2024**(43)	Short-term (1 and 2 years) and long-term (5 years)	Retrospective	Both adjusted and unadjusted effects	No differences in overall survival were observed among children across ethnic groups. Severe acute GVHD (grade III/IV) was higher in Black children. Receiving UCB units from donors of the same race or ethnicity did not improve survival among children. Neighborhood poverty and insurance type are associated with high treatment-related mortality across all racial groups.

Table S1: Summary of the study characteristics.

**Studies**	**Effect of small studies**	**Standard error of effect**	**p-value (Egger's test)**
**Relapse Rate (Blacks vs. Whites)**	3.20	3.871	0.4084
**Relapse Rate (Hispanics vs. Blacks)**	-12.25		0.4029
**Transplantation Rate (Hispanics vs. Blacks)**	2.26	1.141	0.0473

Table S2 . Publication bias was evaluated on comparisons yielding significant associations only.

Study (Author and Year)	Hispanics vs. Whites	Asians vs. Whites	Blacks vs. Whites	Hispanics vs. Blacks
		**Transplantation Rate**		
**Baker et al., 2005**				
**Aplenc et al. (CCG 2891), 2006**	0.925	0.778	0.001	0.003
**Aplenc et al. (CCG 2961), 2006**	0.03	0.819	<0.001	0.037
**Ballen et al., 2012**	0.228		0.403	0.737
**Patel et al., 2015**	<0.001	<0.001	0.688	0.013
**Gramatges et al., 2017**	0.171			
**Blue et al., 2023**	0.001	0.425	0.959	0.024
**Ballen et al. (Adults), 2024**				
		**Relapse Rate**		
**Baker et al., 2005**				
**Aplenc et al. (CCG 2891), 2006**	0.561	0.875	0.089	0.077
**Aplenc et al. (CCG 2961), 2006**	0.725	0.835	0.030	0.038
**Ballen et al., 2012**	0.306		0.603	0.677
**Gramatges et al., 2017**	0.347			
**Blue et al., 2023**	0.029	0.915	0.431	0.029
**Ballen et al. (Adults), 2024**	0.046	0.360	0.830	
**Ballen et al. (Pediatrics), 2024**	0.140	0.990	0.150	
		**Overall Survival**		
**Baker et al., 2005**	0.528	0.134	0.590	0.890
**Aplenc et al. (CCG 2891), 2006**	0.375	0.599	0.316	0.630
**Aplenc et al. (CCG 2961), 2006**	0.503	0.994	0.683	0.930
**Ballen et al., 2012**	0.405		0.177	0.090
**Gramatges et al., 2017**	0.008			
**Blue et al., 2023**	0.001	<0.001	<0.001	0.500
**Ballen et al. (Adults), 2024**	0.500	0.590	0.900	
**Ballen et al. (Pediatrics), 2024**	0.390	0.510	0.025	

Table S3: P values of individual studies

Transplantation Rate						
	**N**	**OR**	**95% LCL**	**95% UCL**	**p-value**	**I^2**
**Hispanics vs. Whites (reference) (Figure 3A)**	6	0.91	0.71	1.16	0.437	85.7%
**Blacks vs. Whites (reference) (Figure 3B)**	5	0.78	0.59	1.03	0.08	77.2%
**Asians vs. Whites (reference) (Figure 3C)**	4	1.17	0.89	1.53	0.254	72.7%
**Hispanics vs. Blacks (reference) (Figure 3D)**	5	1.31	0.90	1.92	0.163	82.4%
**Relapse Rate**						
	**N**	**RR**	**95% LCL**	**95% UCL**	**p-value**	**I^2**
**Hispanics vs. Whites (reference) (Figure 4A)**	7	1.04	0.88	1.24	0.626	51.5%
**Blacks vs. Whites (reference) (Figure 4B)**	6	1.17	1.04	1.32	**0.008**	0.00%
**Asians vs. Whites (reference) (Figure 4C)**	5	1.05	0.86	1.29	0.622	0.00%
**Hispanics vs. Blacks (reference) (Figure 4D)**	4	0.77	0.61	0.97	**0.027**	37.3%
**Overall Survival**						
	**N**	**RR**	**95% LCL**	**95% UCL**	**p-value**	**I^2**
**Hispanics vs. Whites (reference) (Figure 5A)**	8	0.92	0.66	1.27	0.601	70.7%
**Blacks vs. Whites (reference) (Figure 5B)**	7	0.88	0.62	1.26	0.487	70.8%
**Asians vs. Whites (reference) (Figure 5C)**	6	0.95	0.58	1.55	0.827	67.7%
**Hispanics vs. Blacks (reference) (Figure 5D)**	5	1.22	0.90	1.67	0.197	0.0%

Table S4 : Summary of results including all studies

N, Number of studies; OR, Odds ratio; RR, Risk ratio; LCL, Lower confidence limit; UCL, Upper confidence limit; I^2, Heterogeneity

Transplantation Rate						
	**N**	**OR**	**95% LCL**	**95% UCL**	**p-value**	**I^2**
**Hispanics vs. Whites (reference)**	**4**	0.92	0.61	1.38	0.67	81.3%
**Blacks vs. Whites (reference)**	**3**	0.45	0.17	1.16	0.098	85.1%
**Asians vs. Whites (reference)**	**3**	1.29	0.94	1.77	0.111	24.1%
**Hispanics vs. Blacks (reference)**	**3**	2.02	1.15	3.54	**0.014**	53.1%
**Relapse Rate**						
	**N**	**RR**	**95% LCL**	**95% UCL**	**p-value**	**I^2**
**Hispanics vs. Whites (reference)**	**4**	1.04	0.87	1.24	0.658	8.9%
**Blacks vs. Whites (reference)**	**3**	1.33	1.12	1.58	**0.001**	0.0%
**Asians vs. Whites (reference)**	**3**	0.96	0.68	1.37	0.835	0.00%
**Hispanics vs. Blacks (reference)**	**2**	0.70	0.54	0.90	**0.006**	0.00%
**Overall Survival**						
	**N**	**RR**	**95% LCL**	**95% UCL**	**p-value**	**I^2**
**Hispanics vs. Whites (reference)**	**5**	0.84	0.52	1.36	0.487	54.8%
**Blacks vs. Whites (reference)**	**4**	1.27	0.92	1.75	0.149	10.7%
**Asians vs. Whites (reference)**	**4**	1.45	0.92	2.27	0.111	0.0%
**Hispanics vs. Blacks (reference)**	**3**	1.10	0.61	1.98	0.758	0.0%

Table S5 : Summary of results excluding studies with only older patients

N, Number of studies; OR, Odds ratio; RR, Risk ratio; LCI, Lower confidence limit; UCL, Upper confidence limit; I^2, Heterogeneity

Transplantation Rate						
	**N**	**OR**	**95% LCL**	**95% UCL**	**p-value**	**I^2**
**Hispanics vs. Whites (reference)**	3	1.002	0.72	1.40	0.993	93.2%
**Blacks vs. Whites (reference)**	3	0.98	0.89	1.08	0.672	0.0%
**Asians vs. Whites (reference)**	2	1.24	0.89	1.72	0.200	90.1%
**Hispanics vs. Blacks (reference)**	3	1.03	0.75	1.42	0.844	80.6%
**Relapse Rate**						
	**N**	**RR**	**95% LCL**	**95% UCL**	**p-value**	**I^2**
**Hispanics vs. Whites (reference)**	3	1.03	0.73	1.45	0.856	77.9%
**Blacks vs. Whites (reference)**	2	1.05	0.89	1.23	0.552	0.0%
**Asians vs. Whites (reference)**	3	1.10	0.86	1.40	0.46	0.0%
**Hispanics vs. Blacks (reference)**	2	0.84	0.50	1.40	0.498	72.2%
**Overall Survival**						
	**N**	**RR**	**95% LCL**	**95% UCL**	**p-value**	**I^2**
**Hispanics vs. Whites (reference)**	4	1.02	0.67	1.56	0.912	80.6%
**Blacks vs. Whites (reference)**	4	0.80	0.56	1.15	0.223	71.0%
**Asians vs. Whites (reference)**	3	0.84	0.43	1.63	0.611	84.3%
**Hispanics vs. Blacks (reference)**	3	1.22	0.89	1.67	0.22	0.0%

Table S6 : Summary of results excluding studies with only pediatric patients

N: Number of studies; OR, Odds ratio; RR: Risk ratio; LCL: Lower confidence limit; UCL: Upper confidence limit; I^2= Heterogeneity

Supplemental Figures

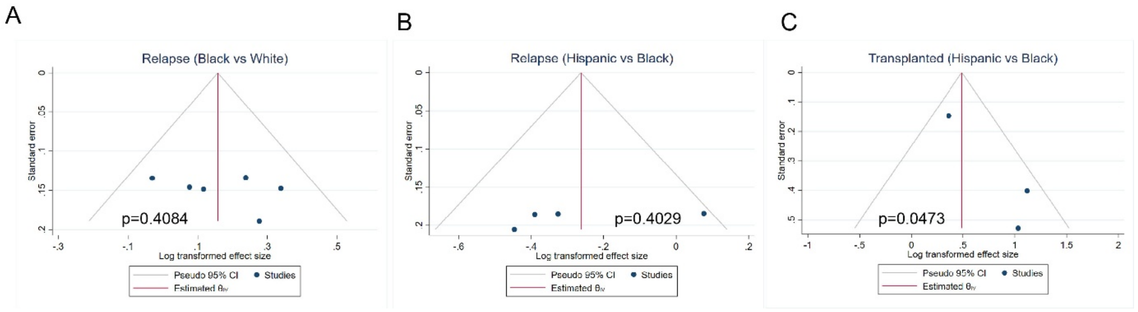

Figure S1. Funnel plots demonstrate publication bias in transplantation rate but not relapse rate reported in the present study. A-C. Funnel plots demonstrate the absence of publication bias comparing relapse of Black vs. White AML patients undergoing hematopoietic stem cell transplantation (HSCT) (**A**) and comparing Hispanic vs. Black AML patients (**B**). Publication bias was present in the study comparing transplantation rates between Hispanics and Blacks (**C**).
